# Work Preferences in Rural Health Job Posting Among Medical Interns in a Lower Middle-Income Country—a Discrete Choice Experiment

**DOI:** 10.5195/cajgh.2020.344

**Published:** 2020-03-31

**Authors:** Julius R. Migriño

**Affiliations:** 1 San Beda University College of Medicine, San Miguel, Manila, Philippines; 2 Ateneo School of Medicine and Public Health, Pasig City, Philippines; 3 University of the Philippines—Open University, Los Baños, Laguna, Philippines

**Keywords:** Global health, Rural job posting, Health human resources, Health systems, Recruitment, Preferences

## Abstract

**Introduction:**

Timely empirical evidence is important in the success of health systems, and such evidence is necessary for informed policy making to address inequity in the health workforce. Literature is ripe with incentives that affect recruitment and retention of physicians in rural and remote areas, but such data in still lacking in the Philippine setting. Discrete choice experiment is one methodology utilized by the World Health Organization which provides both qualitative and quantitative information to aid policy makers in health human resource management.

**Methods:**

The study utilized a discrete choice experiment involving three phases: 1) identification of incentives and levels using key informant interviews and focus group discussions, 2) selection of scenarios utilizing an experimental design, and 3) administration of survey based on WHO guidelines. Conditional logistic regression, point estimates, and correlational analyses were done using Stata.

**Results:**

There is significant association between type of background and considerations for rural practice among the respondents based on Pearson's correlation (p < 0.01). The respondents put more value into non-wage rural job posting incentives than small to modest base salary increases. The high willingness to pay for the presence of supervision, relative location of work areas from families, and status of workplace infrastructure/equipment or supplies suggest the importance of workplace conditions to attract rural health physicians. Combinations of wage and non-wage incentives may be necessary to provide for the most cost-efficient increases in rural job post uptake rates based on post-estimate calculations.

**Conclusion:**

Philippine medical interns and young doctors value non-wage incentives in considering rural health job postings. Rural health job postings with these incentives are predicted to significantly increase recruitment in rural health job posts, particularly when combinations of wage and high-impact non-wage incentives are considered.

Human Resource for Health (HRH) development is vital in the success of a health system, and its success depends on empirical evidence for informed policy making.^1^ There is sufficient evidence of an overall scarcity of health workers globally,^2^^–^^5^ and this is compounded by inequitable distribution of health care workers between urban and rural/remote areas of the world. In the Philippines, where more than half of the population live in rural and remote areas of the country, the density of doctors situated in primarily urban areas such as in the National Capital Region (NCR) and Southern Tagalog are higher compared to some rural and remote provinces in Mindanao and Western and Eastern Visayas, and many health sector positions in rural and isolated areas remain vacant.^6^,^7^ This inequity of distribution and failure to retain health workers in rural areas reduces the population's access to much needed health services, resulting in poor health outcomes such as higher infant mortality rates (IMR): for instance, Eastern Visayas, Western Mindanao, and Autonomous Region of Muslim Mindanao have higher IMR (31, 32, 33 per 1,000 live births, respectively) compared with the national average (23 per 1,000 live births).^8^

One way to address this issue is for policy-makers to have access to information on health worker preference models. There is limited data on assessments of influencing factors or effective strategies to address this gap in human health resources,^9^ including within the setting of the Philippines.^6^,^10^,^11^ Currently, the few preference studies regarding medical practitioners in the Philippines rely on descriptive methods such as cross-sectional surveys and case studies, and do not take into consideration the hierarchy of preferences as well as possibilities of trade-off between difference choices. The discrete choice experiment (DCE) is one method that could be used to quantitatively assess the importance of individual factors which influence health worker preferences to specific job posting incentives. Data from DCEs may aid policy makers in prioritizing rural post incentive packages that would prove to be most cost-effective and have higher uptake potential.

The study aimed to determine the association of different job incentives and the probability of take-up of rural health job postings among medical interns and recent graduates in the Philippines using a discrete choice experiment. The study null hypothesis was that there was no significant association between the presence of different job incentives and the probability of take-up of rural health job postings among medical interns.

## Methods

The study employed a mixed methods discrete choice experiment methodology involving three phases:

Phase 1– Identification of incentives and levels
Phase 2– Selection of scenarios
Phase 3– Administration of survey

The Association of Philippine Medical Colleges Foundation, Inc. (APMCFI) is a non-stock, non-profit organization that “defines standards and guidelines to promote quality medical education in the Philippines” and serves as the umbrella organization of all accredited medical schools in the country.^12^ A complete enumeration of all the listed medical interns was conducted to enable representation of medical interns from all regions of the Philippines. All 7,178 medical students who participated in the medical internship matching system for Academic Years 2016-2017 and 2017-2018 under APMCFI were included in the sampling frame. The inclusive years used in the sampling coincide with the time frame of the study. Sample size was calculated using OpenEpi to be 365 at 95% confidence level, and simple random sampling was done to recruit the DCE respondents.

### Phase 1: Identification of incentives and levels

A literature review was done on incentives to attract and retain health workers in the rural setting, based on the recommendation by WHO.^13^ Interviews with key informants based on recommendations from the WHO report “Increasing access to health workers in remote and rural areas through improved retention”^14^ were done. At the end of the key informant interviews (KII), a list of 21 feasible and relevant job incentives and possible levels was identified.

Two separate focus group discussions (FGD) were conducted with five medical interns and five recently graduated medical doctors. The participants for each FGD were purposively selected based on sex (at least one of each sex was represented per FGD group), current enrollment status as medical interns (for FGD 1), and newly graduated doctors (within 3 years of the study); these respondents closely resemble the final study respondents. Defining of incentives and recategorization of similar incentives were also done during the FGD. The respondents were then asked to select a final set of incentives and to identify different levels for each that are realistic and appropriate in the local context. For example, when discussing salary, participants were asked what they thought was a fair and realistic salary level for the job posting. Each FGD lasted between 1-1.5 hours and was facilitated and transcribed by the researcher. At the end of the FGD, a final list of seven incentives with corresponding levels was generated.

### Phase 2: Selection of scenarios

Data gathered from Phase 1 was used to construct the rural health job postings (“choice sets”). The experimental choice set design was generated using R version 3.4.2 (2017-09-28), using the package AlgDesign and the function optFederov,^15^ and analyzed for orthogonality using PSPP GNU General Public License (version 3, 29 June 2007). This produced an orthogonal array with level balance, minimal overlap, and D-efficiency = 0.926, with minimum collinearity. A systematic level change of the original design^16^ was then employed in generating the alternative choice set design to ensure a higher efficiency.^17^,^18^ At the end of Phase 2, a final Google Form survey questionnaire with 13 choice sets and an embedded informed consent form was constructed.

### Phase 3: Administration of survey

The link to the Google form questionnaire and informed consent was distributed through blind carbon copy emails to each survey respondent. Responses were collected within a 4-week period. Follow-up emails were made after five and ten working days.

Manual domain analysis was done to the data gathered in Phase 1, using the procedure demonstrated by Atkinson and Abu El Haj^19^ using the qualitative data analysis software QDA Miner Lite (v2.0.2). The creation of scenarios in Phase 2 was based on the data from Phase 1 and was performed using orthogonal design from R software version 3.4.2, which generated an orthogonal design for the choice sets that were used in Phase 3.

Coding of Phase 3 data was done using a stacked-format initial data matrix. Univariate analysis (measures of central tendencies and percentages) were done for the demographic profile using Microsoft Excel. Chi square analysis, conditional logistic regression analysis and computations for willingness to pay, changes in uptake rates, and disaggregation of subgroups were done using Stata v.13.0, with the aid of a consultant statistician and using the guidelines set by WHO (2012). Conditional logistic regression analysis was done using the syntax *clogit CHOICE wage equipment supervision family QoL CPD career const, group(obsid)*, with *wage* as a continuous variable and the rest as dummy-coded variables. The value of *CHOICE* refers to either 0 (job post A) or 1 (job post B) as the respondent's choice, while the term *group(obsid)* is the paired observation per choice set. This function assumed a logit model with the probability of choosing job *i* defined as:


$$P_{i}=\frac{\left(\exp(V_{i}\right)}{\sum_{j-\text{l}}^{N}\exp(V_{j})}\;V_{\text{i}/\text{j}}=\text{deterministic}\;\text{utility}\;\text{of}\;\text{posts}\;i/j$$

Willingness to pay was calculated as the ratio of the value of the coefficient of interest to the negative of the cost attribute,


$$WTP_{n}=\;-\;\frac{\partial U{/}_{\partial_{n}}}{\partial U{/}_{\partial_{wage}}}=\frac{\beta_{n}}{\beta_{wage}},$$

with *n* being the incentive of interest.

Changes in uptake rates (with corresponding confidence intervals) were calculated using the *nlcom* command in Stata using the following syntax:


$$\begin{array}{c} nlcom(exp(\_b{\left[wage\right]}^{*}36000+\_b[n])-\\ exp(\_b{\left[wage\right]}^{*}36000))/exp(\_b{\left[wage\right]}^{*}36000)+exp(\_b[\\ wage{]}^{*}36000+\_b[n])),\text{where}\;n=\text{incentive}\;\text{of}\;\text{interest}. \end{array}$$

Calculations for the disaggregation of subgroups were done using the *clogit* function (as above) but utilizing interaction terms. All calculations were done at 95% confidence level.

## Results

There was a total of 345 respondents from the survey, which represents 4.81% of the total population. Table 1 presents a summary of the demographic characteristics of the DCE respondents. The respondents were distributed across multiple regions of the Philippines; however, the majority of the respondents (66.38%) came from schools in Luzon, with 48.70% coming from the Greater Manila Area. Visayas-based respondents made up 24.35%, while those from Mindanao accounted for 9.27% of the total participants. The mean age of the respondents was 26.42 (±2.26) years old, which is the usual age when medical students in the Philippines finish their medical school and take up medical internship. Most of the respondents were female (68.12%), and a significant majority (93.33%) were single. The general trend in the family income of the respondents belong to middle- and upper-class income brackets. Seventy-two percent of the respondents have lived mostly in urban environments, while a majority (68.12%) at least considered practicing in a rural area after graduation, whether as *Doctors to the Barrios* (DTTB), Municipal Health Officers (MHO), or in rural private practice. Pearson's chi square results revealed a significant difference (p<0.01) between the type of background in relation to considerations of practicing medicine in rural areas.

**Table 1.** Demographic characteristics of DCE respondents (N=345)

Variable	N (%)
**Age** (mean(std. dev.))	26.42 (2.26)
**Sex**	
Male	110 (31.88)
Female	235 (68.12)
**Marital status**	
Single	322 (93.33)
Married	23 (6.67)
**Family's monthly income (in PHP)**	
less than 9,000	8 (2.32)
9.000 to less than 17,000	8 (2.32)
17,000 to less than 35.000	47 (13.62)
35,000 to less than 125,000	148 (42.90)
125.000 to less than 185.000	40 (11.59)
185.000 and above	94 (27.25)
**Living environment background**	
Rural	96 (27.83)
Urban	249 (72.17)
**Location of attended medical school**	
Luzon	229 (66.38)
Visayas	84 (24.35)
Mindanao	32 (9.27)
Greater Metro Manila	168 (48.70)
**Considering working in a rural area soon after graduation?**	
Yes	235 (68.12)
No	110 (31.88)

Based on the directly stated preferences for all identified job incentives (“Which of the following is the MOST IMPORTANT factor in your decision to work in a rural area?”, results not shown), career and distance of family from work facility was ranked as the most important, which is consistent with the results of the conditional logit model.

Main effects conditional logistic regression analysis produced coefficients that were consistent with what was expected: the respondents preferred job postings with higher salaries (“wage”), available equipment (“equipment”), presence of supervision (“supervision”), locations near their families’ residences (“family”), better quality of life amenities (“QoL”), and available continuing professional development (CPD; “CPD”) and career opportunities (“career”). All seven incentives included in the model were significant at 1% level.

**Figure 1.**
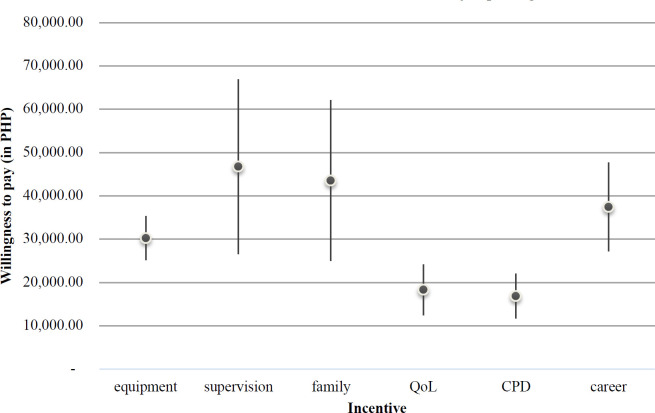
Willingness to pay (WTP) for specific incentives, with 95% confidence intervals

Figure 1 shows how much (in Philippine currency, PHP) the respondents are willing to trade off from their monthly salary to get other incentives in the job package. The most significant willingness to pay (WTP) are those for “supervision” (PHP46,720.38, 95% CI: 26,512.59-66,928.16) and “family” (PHP43,467.51, 95% CI: 24,919.09-62,015.92), with both incentives having wide confidence intervals. WTP_career_ came third (PHP37,433.40, 95% CI: 27,207.71-47,659.09). As for the status of equipment in a rural job post, WTP_equipment_ ranks fourth (PHP30,202.40, 95% CI: 25,104.13-35,300.67), but respondents have less variation with this incentive. “QoL” and “CPD” have consistently low WTP values (PHP18,281.89, 95% CI: 12,401.89-24,161.89; PHP16,850.27, 95% CI: 11,688.33-22,012.22, respectively), stating that compared to the other incentives, the respondents are not very much concerned with the availability of daily living amenities and CPD incentives in rural job postings.

**Figure 2.**
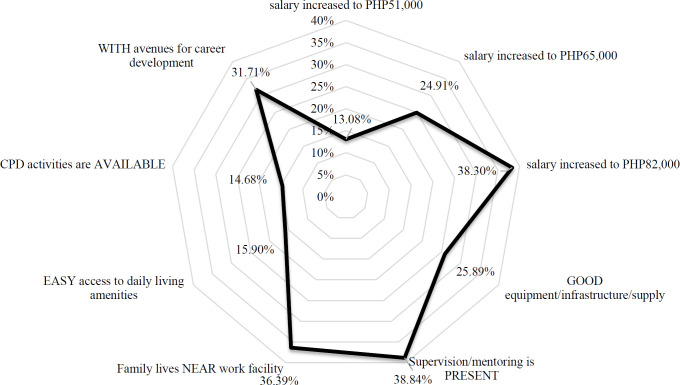
Uptake rates for selected job incentives compared to baseline, in % (N=345)

Note: all uptake rates are significant at 1% level. Baseline job posting incentives include base salary PHP36,000, poor equipment, absent supervision, family far from work, poor living amenities, CPD unavailable, and no avenues for career development.

The relative effectiveness of different policy options was forecasted by calculating for differences in uptake rates compared with the worst-case (“baseline”) job posting scenario: base salary PHP36,000, poor equipment, absent supervision, family far from work, poor living amenities, CPD unavailable, no avenues for career development. Using random utility theory discussed in most health-related DCE and econometric studies which states that respondents will select the option with the highest utility relative to the other choices,^13^,^20^,^21^ uptake rates of the baseline job post with a change in level of each of the incentives were calculated (Figure 2). Among the non-wage incentives, presence of supervision was the most valued incentive (uptake rate=38.84%, SE 0.040), estimated to produce an impact a little above what could be expected from a 228% increase in base salary. This is followed by the impact of work facilities being near a physician's family (36.39%, SE 0.040), then by provisions for career development (31.71%, SE 0.016).

**Figure 3.**
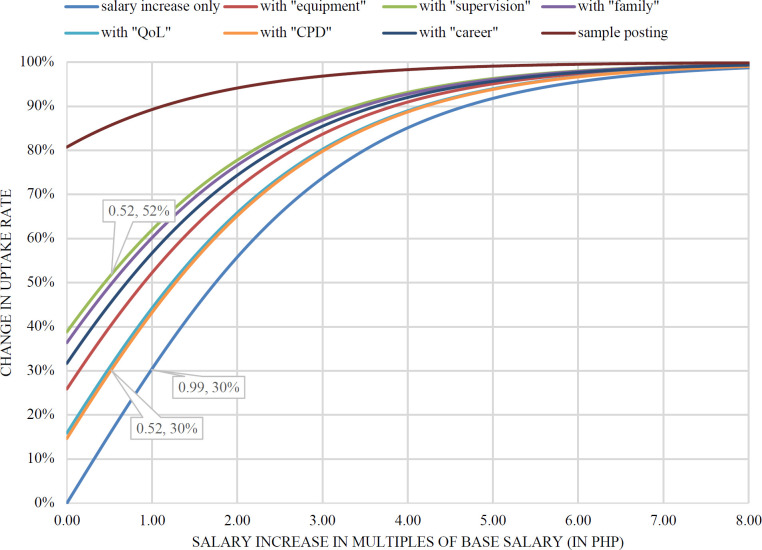
Changes in uptake rates with different job incentives as base salary increases

Note: Sample posting= with “supervision”, “family”, “career”

Post-estimation analysis (Figure 3) shows the relationship between increases in salary with the estimated uptake probabilities: with a 100% increase in salary alone, uptake rates may increase by 30%. However, a modest (52%) increase in salary *plus* ensuring provisions of avenues for CPD may provide a similar increase in uptake rates; this is worth noticing since “CPD” has been identified as a low-impact incentive in this study. Conversely, providing for a high-impact incentive such as supervision in the workplace may net a perceptible increase (52%) in uptake rates, given the same modest (52%) increase in salary. Based on all the permutations presented, it can also be observed that at a certain level, further increases in wage led to decreasing net changes on uptake rates; at a certain level of wage increase, combinations of non-wage incentives might net higher uptake rates. The “sample posting” graph shows the impact of a combination of the three high-impact non-wage incentives (“supervision”, “family”, and “career”) with respect to changes in base salary.

## Discussion

The demographic profile of the respondents closely resembled the general characteristics of medical interns in the Philippines. The greater distribution of the respondents to the Greater Manila Area reflect the location of most medical schools in the Philippines. Low response rates are consistent with electronic survey forms;^22^ however, Johnson et al. states that precision of DCE studies “flattens out at around 300 observations”,^19^ while Lemiere mentions that DCE studies only need a small (~100) effective sample size.^23^ Due to the nature of the survey, it should be noted that the final sample population may not be fully representative of the target population. Yun and Trumbo have shown that internet-based surveys tend to favor males of above-average socioeconomic backgrounds;^22^ these finding were not consistent with the result of the study.

Medical students’ and graduates’ considerations to practice in rural areas rely in part on their rural upbringing: multiple studies have investigated the role of rural vs urban backgrounds in a young physician's decision to pursue rural practice.^24^^–^^27^ In fact, a local study by Leonardia et al. confirms that a larger cohort of DTTBs came from rural backgrounds.^28^ Similar analysis for either sex or marital status as showing associations with doctors’ considerations of work opportunities in rural/urban areas were not significant. The result for directly stated preferences for all identified job incentives was consistent with past studies involving both doctors^9^,^20^,^29^ and nurses,^30^ but most studies on career involve career advancement after serving a specified time in rural practice as opposed to concurrent career advancement identified in this study. Additionally, the conditional logistic regression analysis results cross-validate the results of the qualitative phase of the study and are also consistent with previous DCE studies.^9^,^20^,^21^,^30^^–^^32^

Presence of supervision has been identified as a major factor in both qualitative and quantitative studies regarding rural job post preferences,^20^,^30^,^32^ and this can be triangulated from comments in Phase 1 of this study. It should also be noted that the incentive “family” is not routinely seen in other DCE studies; usually, location of the work area is relative to the nearest city.^20^,^32^ Only one DCE study was found to include interaction terms for “family”: the study by Smitz et al. in Timor-Leste noted significantly higher uptake rates of doctors with rural-based families compared with those with urban-based ones.^21^ This should be considered in estimating the importance of this incentive in policy exercises. There also appeared to be a discrepancy with the relative importance of supervision: it ranked #5 when respondents were asked the directly stated preference question, but the DCE model concluded high β_supervision_ (0.82) and WTP_supervision_ (PHP46,720.38), consistent with previous studies^9^,^20^ where some degree of supervision is among the most significant incentives in rural practice. This discrepancy proves the utility of DCE to capture preferences not routinely seen in conventional stated preference methodologies.^23^

Kolstad validates the inclusion of “career”, where her study in Tanzania confirms the relative importance of professional development early in the careers of young physicians;^20^ however, this study highlights the greater relative importance of supervision compared with career advancement, which was emphasized in another study by Kruk et al. in Ghana.^9^ Additionally, this study, a study by Rana et al. in Pakistan, and a study by Hanson and Jack in Ethiopia confirmed the importance of equipment status as a main consideration in rural job posts,^29^,^32^ where respondents mentioned that poor equipment/infrastructure status and availability of supplies gives “a sense of unproductivity” which may lead to frustration.^29^

The use of DCE enables researchers to minimize the major pitfall of other stated preference models, which is manipulation of responses due to direct or indirect incentives for the respondents.^20^ Using fixed effects models such as was done in this study, including that for salary (“wage”), is common practice; however, it may lead to implausible mean WTP estimates^33^ as was evident in the unrealistically high mean WTP of most incentives. However, in such cases the WTP values should be interpreted relative to each other than as absolute values to provide a more realistic impact of each incentive.^31^

Overall, the post-estimation model predicts better uptake rates for job postings by including any one of the non-wage incentives listed here compared to a moderate increase in base salary. The studies by Kruk et al.,^9^ Kolstad^20^ and Serneels et al.,^34^ as well as by Smitz et al.,^21^ showed similar results, where uptake rates from provision of non-wage incentives such as better equipment status or presence of supervision were comparable to uptake rates from modest to significantly high increases in salary. These studies explained such results by the respondents’ high intrinsic motivation, relatively higher paying jobs, and/or being at the early stages of their professional career.^21^

The study also tried to predict the impact of increases in wage (base salary) together with specific non-wage improvements in rural job postings. Increasing base salary or giving monetary incentives have been identified as “the most obvious way to induce greater labor supply”^32^ and results from the KIIs and FGDs in this study back up this argument (“higher base salary may offset the effect of absence of non-wage incentives”); however, estimates of increasing base salary by itself might not be the most efficient way to increase uptake rates, particularly from early-career doctors.

Due to relatively low levels of financial motivation from recent medical graduates in consideration of rural practice, the Department of Health (DOH) and local government units (LGUs) may focus on adding non-wage incentives to current packages to produce a significant effect on uptake rates, taking into consideration the significant impact of a new doctor's rural upbringing, background, and experience to consideration of the opportunity to practice medicine in rural communities. Cost estimates of potentially high-impact non-wage incentives at the LGU level should also be conducted to see if such incentives are financially feasible. Policy makers such as officers of the DOH-Health Human Resource Development Bureau and MHOs need to evaluate the cost-effectiveness of combinations of incentives from current rural job posts, as well as in the design of new rural health job posts. This could help increase uptake rates of vacant rural health job posts, improve health indices and even help in the prioritization of the health budget of an LGU. In the selection process for medical students, the study's disaggregated data (results not shown) signify the need for rural backgrounds and/or rural community experiences to be given higher priority. However, since DCE utilizes stated preferences, and few studies have been done to determine the relationship of stated preference and revealed preference models, it is highly recommended that routine monitoring of actual physician choices be done to validate the utility of models from similar studies using longitudinal cohort studies.

## References

[1] Diallo K, Zurn P, Gupta N, Dal Poz M. Monitoring and evaluation of human resources for health: an international perspective. *Hum Resour Health*. 2003;1(1):3. doi: 10.1186/1478-4491-1-3. 12904252 PMC179874

[2] WHO. WHO | The World Health Report 2006 - working together for health. https://www.who.int/whr/2006/en/. Accessed July 29, 2020.

[3] Badr E, Mohamed NA, Afzal MM, Bile KM. Strengthening human resources for health through information, coordination and accountability mechanisms: the case of the Sudan. *Bull World Health Organ*. 2013;91(11):868–73. doi: 10.2471/BLT.13.118950. 24347712 PMC3853958

[4] Packer C, Labonte R, Spitzer D. WHO Commission on Social Determinants of Health: Globalization and Health Worker Crisis. Globalization Knowledge Network. http://www.who.int/social_determinants/resources/gkn_packer_al.pdf.. Published 2007. Accessed July 29, 2020.

[5] WHO. Global strategy on human resources for health: Workforce 2030. World Health Organization. https://www.who.int/hrh/resources/global_strategy_workforce2030_14_print.pdf.. Published 2016. Accessed July 29, 2020.

[6] Romualdez A, dela Rosa JF, Flavier JD, et al. The Philippines Health System Review. Manila, Philippines: World Health Organization, Western Pacific Region. http://www.searo.who.int/entity/asia_pacific_observatory/publications/hits/hit_philippines/en/.. Published 2011. Accessed July 29, 2020.

[7] WHO, DOH. Health Service Delivery Profile - Philippines 2012. http://www.wpro.who.int/health_services/service_delivery_profile_philippines.pdf.. Published 2012.

[8] Philippine Statistics Authority. PSA-i-MDGs-Table: Infant Mortality Rate by Region and Year. http://nap.psa.gov.ph/iMDGs/PX/Dialog/varval.asp?ma=4bvinimr&ti=Infant+Mortality+Rate+by+Region+and+Year%2E+&path=../Database/NSCB/Goals/Goal4/&lang=1&unit=Index. Accessed 2019 Jan 12.

[9] Kruk ME, Johnson JC, Gyakobo M, et al. Rural practice preferences among medical students in Ghana: a discrete choice experiment. *Bull World Health Organ*. 2010;88(5):333–41. doi: 10.2471/BLT.09.072892 20458371 PMC2865662

[10] Ronquillo K, Elegado-Lorenzo F, Nodora R. Human Resources for Health Migration in the Philippines: A Case Study and Policy Directions. *ASEAN Learning Network for Human Resources for Health*. 2005;August 2-5.

[11] Institute of Health Policy and Development Studies. Migration of health workers: Country case study Philippines. http://www.ilo.org/sector/Resources/publications/WCMS_161163/lang--en/index.htm.. Published 2005. Accessed July 29, 2020.

[12] APMCFI. Vision and Mission | Association of Philippine Medical Colleges Foundation Inc. (APMCFI). Association of Philippine Medical Colleges Foundation, Inc. http://webv2.apmcfph.net/apmc_wp/about-apmc/vision-and-mission/.. Published 2016. Accessed July 29, 2020.

[13] WHO. How to Conduct a Discrete Choice Experiment for Health Workforce Recruitment and Retention in Remote and Rural Areas: A USER GUIDE WITH CASE STUDIES. World Health Organisation. https://www.who.int/hrh/resources/DCE_UserGuide_WEB.pdf.. Published 2012. Accessed July 29, 2020.

[14] Dolea C. Increasing access to health workers in remote and rural areas through improved retention: global policy recommendations. Geneva, Switzerland: World Health Organization; 2010. 72 p. 23741785

[15] Stack Exchange. How to create a nearly orthogonal experimental design in R?. Cross Validated. https://stats.stackexchange.com/questions/110133/howto-create-a-nearly-orthogonal-experimental-design-in-r. Accessed July 29, 2020.

[16] Reed Johnson F, Lancsar E, Marshall D, et al. Constructing experimental designs for discrete-choice experiments: report of the ISPOR Conjoint Analysis Experimental Design Good Research Practices Task Force. *Value Health*. 2013;16(1):3–13. doi: 10.1016/j.val.2012.08.2223 23337210

[17] Street DJ, Burgess L, Louviere JJ. Quick and easy choice sets: Constructing optimal and nearly optimal stated choice experiments. *International Journal of Research in Marketing*. 2005;22(4):459–70. doi: 10.1016/j/ijresmar.2005.09.003.

[18] Tang L, Luo X, Cheng Y, Yang F, Ran B. Comparing the State-of-the-Art Efficient Stated Choice Designs Based on Empirical Analysis. *Mathematical Problems in Engineering*. 2014. doi: 10.1155/2014/740612

[19] Atkinson S, Abu el Haj M. Domain analysis for qualitative public health data. *Health Policy Plan*. 1996;11(4):438–42. doi: 10.1093/heapol/11.4.438 10164200

[20] Kolstad JR. How to make rural jobs more attractive to health workers. Findings from a discrete choice experiment in Tanzania. *Health Econ*. 2011;20(2):196–211. doi: 10.1002/hec.1581 20094993

[21] Smitz M-F, Witter S, Lemiere C, et al. Understanding Health Workers' Job Preferences to Improve Rural Retention in Timor-Leste: Findings from a Discrete Choice Experiment. *PLOS ONE*. 2016;11(11). doi: 10.1371/journal.pone.0165940 PMC5112867 27846242

[22] Yun GW, Trumbo CW. Comparative Response to a Survey Executed by Post, E-mail, & Web Form. J Comput Mediat Commun. 2000;6(1). doi: 10.1111/j.1083-6101.2000.tb00112.x

[23] Lemiere C. Discrete Choice Experiment (DCE): a Methodology for Eliciting Health Workers' Preferences. World Health Organization; 2009. Available from: https://www.hrhresourcecenter.org/node/2424.html. Accessed July 29, 2020.

[24] Chan BTB, Degani N, Crichton T, et al. Factors influencing family physicians to enter rural practice: does rural or urban background make a difference?. *Can Fam Physician*. 2005;51(9):1247. https://pubmed.ncbi.nlm.nih.gov/16926939. Accessed July 29, 2020. PMC1479469 16926939

[25] McGrail MR, Humphreys JS, Joyce CM. Nature of association between rural background and practice location: A comparison of general practitioners and specialists. BMC Health Serv Res. 2011;11(1):63. doi: 10.1186/1472-6963-11-63 21429224 PMC3074548

[26] Rodriguez K. Literature Review of Recruitment and Retention for Rural Health Care Providers. Maine AHEC Network. https://www.une.edu/sites/default/files/%281%29%20Rural%20Physician%20Recruitment%20and%20Retention%20Lit%20Review%20FINAL%5B1%5D.pdf.. Published 2014. Accessed July 29, 2020.

[27] Serneels P, Montalvo JG, Pettersson G, Lievens T, Butera JD, Kidanu A. Who wants to work in a rural health post? The role of intrinsic motivation, rural background and faith-based institutions in Ethiopia and Rwanda. *Bull World Health Organ*. 2010;88(5):342–9. doi: 10.2471/BLT.09.072728. 20461138 PMC2865659

[28] Leonardia JA, Prytherch H, Ronquillo K, Nodora RG, Ruppel A. Assessment of factors influencing retention in the Philippine National Rural Physician Deployment Program. *BMC Health Serv Res*. 2012;12(1):411. doi: 10.1186/1472-6963-12-411. 23167701 PMC3511213

[29] Rana SA, Sarfraz M, Kamran I, Jadoon H. Preferences Of Doctors For Working In Rural Islamabad Capital Territory, Pakistan: A Qualitative Study. *J Ayub Med Coll Abbottabad*. 2016;28(3):591–596. https://pubmed.ncbi.nlm.nih.gov/28712243. Accessed July 29, 2020. 28712243

[30] Lagarde M, Blaauw D. A review of the application and contribution of discrete choice experiments to inform human resources policy interventions. *Human Resour Health*. 2009;7(1):62. doi: 10.1186/1478-4491-7-62. PMC2724490 19630965

[31] Rockers PC, Jaskiewicz W, Wurts L, et al. Preferences for working in rural clinics among trainee health professionals in Uganda: a discrete choice experiment. *BMC Health Serv Res*. 2012;12:212. doi: 10.1186/1472-6963-12-212. 22824497 PMC3444383

[32] Hanson K, Jack W. Health worker preferences for job attributes in Ethiopia?: results from a discrete choice experiment. Washington, D.C: World Bank Group. http://documents.worldbank.org/curated/en/716191468030240068/Health-worker-preferences-for-job-attributes-in-Ethiopia-results-from-a-discrete-choice-experiment.. Published April 2008. Accessed July 29, 2020.

[33] Hole AR. Estimation of willingness to pay in preference space vs. WTP space. University of Sheffield. https://www.sheffield.ac.uk/polopoly_fs/1.214046!/file/DCMD.pdf.. Published December 8, 2011. Accessed July 29, 2020.

[34] Serneels P, Lindelow M, Montalvo JG, Barr A. For public service or money: understanding geographical imbalances in the health workforce. *Health Policy Plan*. 2007;22(3):128–38. doi: 10.1093/heapol/czm005. 17463013

