# Supplementary material for: Work Preferences in Rural Health Job Posting Among Medical Interns in a Lower Middle-Income Country—a Discrete Choice Experiment
Source: Cent Asian J Glob Health. 2020 Mar 31;9(1):e344. doi: 10.5195/cajgh.2020.344 (PMC9295858; doi:10.5195/cajgh.2020.344)
Supplement: Supplementary file 1 [file cajgh-9-e344-Suppl.docx]

# Title Page

***Work preferences in rural health job posting among medical interns in a lower middle-income country – a discrete choice experiment***

**Julius R. Migriño, Jr., MD, MIH, DPASMAP** – corresponding author

San Beda University College of Medicine – author primary affiliation

Ateneo School of Medicine and Public Health – author secondary affiliation

University of the Philippines – Open University – study affiliation

Phone: +63 925 729 7292

Email: jrmjrmd-1@yahoo.com

# SUPPLEMENTARY TABLES AND FIGURES

Table. Summary of incentives influencing preference for rural health job postings, identified from literature review and KIIs

| **Code** | **Description** |
| --- | --- |
| CPD | avenues for continued medical education (CME) and continuing professional development (CPD) |
| association | avenues for membership in professional associations |
| scope | avenue for increased scope of practice (beyond clinical work, includes other duties such as administration) |
| family | Distance of family from work facility |
| RSA | “Return of Service Agreement”: service memorandum of agreement, |
| wage | salary/base pay (either from DOH or LGU, in PHP) |
| LGU support | LGU support in terms of health programs |
| promotion | possibility and avenues for promotion, either in DOH or LGU (or transfer with promotion) |
| private practice | practice outside of clinical work in DOH or LGU (includes private practice with or without PhilHealth compensation, private businesses, consultancies etc.) |
| equipment | status of equipment, infrastructure, and supplies in work area |
| supervision | presence or absence of supervision/mentoring from DOH, LGU health offices, or specialty associations |
| MPM | post-graduate academic training while in service (e.g. Master’s in Public Management) |
| residency | post-graduate residency training while in service (i.e. Family Medicine) |
| recognition | awards and recognition from LGU/DOH |
| postgrad | support/grants for academic/residency training after service |
| subsistence | daily living allowance, includes clothing and meal allowances |
| travel | representation and travel allowance (RATA) for conventions, meetings, research etc. |
| hazard pay | hazard and other related pay incentives |
| leave | medical and personal leave, with pay |
| housing | provision/subsidy for housing, includes utilities |

Table. Sample of Job Posting Choice Set

| **Job Posting A** | **Job Posting B** |
| --- | --- |
| monthly salary PHP 65,000 | monthly salary PHP 36,000 |
| POOR equipment/infrastructure/supply | GOOD equipment/infrastructure/supply |
| Supervision/mentoring is ABSENT | Supervision/mentoring is PRESENT |
| Family lives NEAR work facility | Family lives FAR from work facility |
| POOR access to daily living amenities | EASY access to daily living amenities |
| CPD activities are NOT AVAILABLE | CPD activities are AVAILABLE |
| WITHOUT avenues for career development | WITH avenues for career development |

Table. Pearson’s chi-square analysis, type of background v considerations to work in rural/urban areas

|  | **Considering URBAN practice** | **Considering RURAL practice** | **Total** |
| --- | --- | --- | --- |
| **URBAN Background** | 95 | 154 | 249 |
|  | (27.54%) | (44.64%) | (72.17%) |
| **RURAL Background** | 15 | 81 | 96 |
|  | (4.35%) | (23.48%) | (27.83%) |
| **Total** | 110 | 235 | 345 |
|  | (31.88%) | (68.12%) | (100.00%) |
|  |  |  |  |
|  | Pearson chi2(1) = 16.1905 | | Pr = 0.000 |

Figure. Frequency count, “which of the following is the MOST IMPORTANT factor in your decision to work in a rural area?” (N=345)

Table. Results of a conditional logit model of DCE data

| **Incentive** | **β coefficient** | **Standard Error** | **p > \|z\|** |
| --- | --- | --- | --- |
| **wage** | 1.75E-05 | 2.31E-06 | 0.000 |
| **equipment** | 0.530 | 0.058 | 0.000 |
| **supervision** | 0.820 | 0.093 | 0.000 |
| **family** | 0.763 | 0.092 | 0.000 |
| **QoL** | 0.321 | 0.047 | 0.000 |
| **CPD** | 0.296 | 0.055 | 0.000 |
| **career** | 0.657 | 0.037 | 0.000 |
| **const** | 0 | (omitted) |  |
|  |  |  |  |
|  |  |  |  |
| **Log likelihood** | -2364.8525 |  |  |
| **Number of obs** | 8280 |  |  |
| **LR chi2(7)** | 1009.55 |  |  |
| **Prob > chi2** | 0.0000 |  |  |
| **Pseudo R2** | 0.1759 |  |  |

Note: all coefficients are significant at 1% level.

Table. Willingness to pay (WTP) for specific incentives

| **Incentive** | **WTP** | **Lower level of 95% CI** | **Upper level of 95% CI** |
| --- | --- | --- | --- |
| **equipment** | 30,202.40 | 25,104.13 | 35,300.67 |
| **supervision** | 46,720.38 | 26,512.59 | 66,928.16 |
| **family** | 43,467.51 | 24,919.09 | 62,015.92 |
| **QoL** | 18,281.89 | 12,401.89 | 24,161.89 |
| **CPD** | 16,850.27 | 11,688.33 | 22,012.22 |
| **career** | 37,433.40 | 27,207.71 | 47,659.09 |
|  |  |  |  |
|  |  |  |  |
| **Number of obs** | 8280 |  |  |
| **LR chi2(7)** | 1009.55 |  |  |
| **Prob > chi2** | 0.0000 |  |  |
| **Pseudo R2** | 0.1759 |  |  |

Note: All values are in PHP per month. Confidence intervals were calculated using STATA’s -nlcom- command.

1 USD = PHP 51.56

Figure. Uptake rates for selected job incentives compared to baseline, in % (N=345)

Notes: all uptake rates are significant at 1% level. Baseline job posting incentives include: base salary PHP36,000, poor equipment, absent supervision, family far from work, poor living amenities, CPD unavailable, no avenues for career development.

Table. Uptake rates per subgroup, in % (Total N=345)

| Incentive | Male  (N=110) | Female  (N=235) | Rural Background  (N=96) | Urban Background  (N=249) | Considering working in a rural area  (N=235) | Considering working in an urban area  (N=110) |
| --- | --- | --- | --- | --- | --- | --- |
| salary increased to PHP51,000 | 13.72% (4.48%) | 12.99% (1.82%) | 11.36% (4.74%) | 13.31% (1.80%) | 12.62% (3.84%) | 13.27% (2.18%) |
| salary increased to PHP65,000 | 25.43% (4.62%) | 24.71% (3.36%) | 23.46% (4.88%) | 25.33% (3.31%) | 24.63% (3.77%) | 25.24% (4.02%) |
| salary increased to PHP82,000 | 38.68% (5.15%) | 38.04% (4.85%) | 37.20% (5.37%) | 38.90% (4.77%) | 38.23% (4.57%) | 38.79% (5.78%) |
| GOOD equipment/ infrastructure/supply | 25.60% (3.64%) | 26.03% (2.95%) | 28.39% (3.76%) | 24.93% (2.92%) | 27.14% (2.93%) | 23.24% (3.67%) |
| Supervision/mentoring is PRESENT | 37.10% (4.58%) | 39.65% (4.06%) | 44.16% (4.42%)^a^ | 36.77% (4.14%)^a^ | 40.35% (4.04%)^c^ | 35.60% (4.64%)^c^ |
| Family lives NEAR work facility | 37.14% (4.55%) | 36.04% (4.14%) | 31.60% (4.86%)^b^ | 38.22% (4.05%)^b^ | 35.49% (4.16%) | 38.30% (4.51%) |
| EASY access to daily living amenities | 15.71% (3.40%) | 15.99% (2.57%) | 12.47% (3.62%) | 17.23% (2.52%) | 14.31% (2.58%) | 19.30% (3.36%) |
| CPD activities are AVAILABLE | 13.67% (3.68%) | 15.15% (2.92%) | 17.92% (3.82%) | 13.43% (2.89%) | 15.95% (2.92%) | 11.95% (3.70%) |
| WITH avenues for career development | 32.07% (2.84%) | 31.54% (1.97%) | 36.60% (2.97%)^b^ | 29.82% (1.93%)^b^ | 32.44% (1.97%) | 30.15% (2.86%) |

Notes: all uptake rates are significant at 5% level unless noted as *. Separate regressions were run for each subgroup using the same baseline (base salary PHP36,000, poor equipment, absent supervision, family far from work, poor living amenities, CPD unavailable, no avenues for career development). Significant differences in preferences between subgroups are as follows: ^a^ significant at 5% level; ^b^ significant at 10% level; ^c^ significant at 15% level. Standard errors are in parentheses ().

Table. ***(continued)*** Uptake rates per subgroup, in % (Total N=345)

| Incentive | SINGLE  (N=322) | MARRIED  (N=23) | | PUBLIC MEDICAL SCHOOL  (N=40) | | PRIVATE MEDICAL SCHOOL  (N=305) | | CLass CDE  (N=211) | | Class AB  (N=134) | |  |
| --- | --- | --- | --- | --- | --- | --- | --- | --- | --- | --- | --- | --- |
| salary increased to PHP51,000 | 0.15% (9.73%)^*^ | | 17.89% (3.90%) | | 20.35% (9.53%) | | 12.83% (1.74%) | | 5.53% (3.88%)*^a^ | | 15.67% (2.06%)^a^ | |
| salary increased to PHP65,000 | 12.05% (10.22%)^*^ | | 33.61% (6.91%) | | 33.47% (11.28%) | | 24.43% (3.21%) | | 11.96% (6.89%)* | | 29.62% (3.73%) | |
| salary increased to PHP82,000 | 33.02% (13.36%) | | 50.40% (9.21%) | | 58.78% (16.77%) | | 37.61% (4.65%) | | 30.23% (10.67%) | | 44.98% (5.18%) | |
| GOOD equipment/ infrastructure/supply | 26.73% (2.75%)^b^ | | 14.20% (6.97%)^b^ | | 27.27% (5.26%) | | 25.71% (2.79%) | | 27.87% (2.99%) | | 22.75% (3.47%) | |
| Supervision/mentoring is PRESENT | 42.25% (3.89%)^a^ | | -7.67% (7.92%)*^a^ | | 53.04% (4.97%)^a^ | | 36.92% (4.06%)^a^ | | 45.07% (3.91%)^a^ | | 28.75% (4.71%)^a^ | |
| Family lives NEAR work facility | 33.61% (4.13%)^a^ | | 71.72% (4.13%)^a^ | | 23.77% (6.33%)^a^ | | 38.02% (3.99%)^a^ | | 30.43% (4.36%)^a^ | | 45.66% (4.09%)^a^ | |
| EASY access to daily living amenities | 16.71% (2.33%)^b^ | | 4.54% (6.97%)*^b^ | | 14.34% (5.28%) | | 16.11% (2.37%) | | 15.26% (2.67%) | | 16.93% (3.14%) | |
| CPD activities are AVAILABLE | 16.02% (2.71%)^a^ | | -4.25% (7.25%)*^a^ | | 19.83% (5.35%) | | 14.00% (2.75%) | | 19.76% (2.97%)^a^ | | 6.52% (3.51%)^a^ | |
| WITH avenues for career development | 31.83% (1.70%) | | 30.04% (6.19%) | | 32.02% (4.66%) | | 31.67% (1.75%) | | 30.98% (2.08%) | | 32.87% (2.58%) | |

Notes: all uptake rates are significant at 5% level unless noted as *. Separate regressions were run for each subgroup using the same baseline (base salary PHP36,000, poor equipment, absent supervision, family far from work, poor living amenities, CPD unavailable, no avenues for career development). Significant differences in preferences between subgroups are as follows: ^a^ significant at 5% level; ^b^ significant at 10% level; ^c^ significant at 15% level. Standard errors are in parentheses ().
